# Case study of CD19 CAR T therapy in a subject with immune-mediate necrotizing myopathy treated in the RESET-Myositis phase I/II trial

**DOI:** 10.1016/j.ymthe.2024.09.009

**Published:** 2024-09-07

**Authors:** Jenell Volkov, Daniel Nunez, Tahseen Mozaffar, Jason Stadanlick, Mallorie Werner, Zachary Vorndran, Alexandra Ellis, Jazmean Williams, Justin Cicarelli, Quynh Lam, Thomas Furmanak, Chris Schmitt, Fatemeh Hadi-Nezhad, Daniel Thompson, Claire Miller, Courtney Little, David Chang, Samik Basu

**Affiliations:** 1 Cabaletta Bio, Philadelphia, PA, USA; 2 University of California Irvine School of Medicine, Department of Neurology, Irvine, CA, USA

## Abstract

Under compassionate use, chimeric antigen receptor (CAR) T cells have elicited durable remissions in patients with refractory idiopathic inflammatory myopathies (IIMs). Here, we report on the safety, efficacy, and correlative data of the first subject with the immune-mediated necrotizing myopathy (IMNM) subtype of IIM who received a fully human, 4-1BBz anti-CD19-CAR T cell therapy (CABA-201) in the RESET-Myositis phase I/II trial (NCT06154252). CABA-201 was well-tolerated following infusion. Notably, no evidence of cytokine release syndrome or immune effector cell-associated neurotoxicity syndrome was observed. Creatine kinase levels decreased, and muscular strength improved post-infusion. Peripheral B cells were depleted rapidly following infusion, and the subject achieved peripheral B cell aplasia by day 15 post-infusion. Peripheral B cells returned at 2 months post-infusion and were almost entirely transitional. Autoantibodies to SRP-9, SRP-72, SRP-54, and Ro-52, decreased relative to baseline, whereas antibodies associated with pathogens and vaccinations remained stable. The infusion product consisted of predominantly CD4^+^ effector memory T cells and exhibited *in vitro* cytolytic activity. Post-infusion, CABA-201 expansion peaked at day 15 and was preceded by a serum IFN-γ peak on day 8 with peaks in serum IL-12p40 and IP-10 on day 15. These data detail the safety, efficacy, and pharmacodynamics of CABA-201 in the first IMNM subject.

## Graphical abstract

**Figure:**
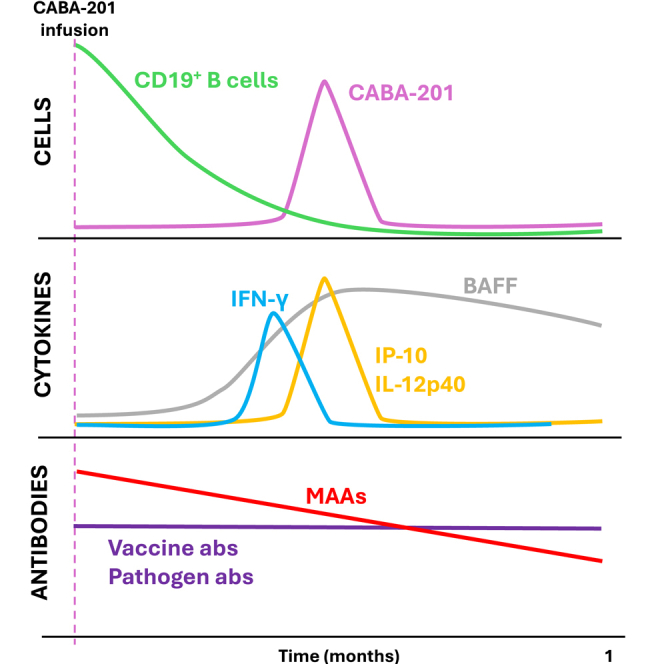

## Teaser

Volkov, Nunez, and colleagues show CABA-201, a human anti-CD19 41BBζ CAR T cell product, rapidly and transiently eliminates B cells in an immune-mediated necrotizing myopathy subject, leading to decreases in serum CK and auto-antibodies and improvements in muscle strength. This is the first reported use of CD19 CAR T cell product in IMNM.

## Introduction

Myositis, also known as idiopathic inflammatory myopathy (IIM), encompasses a heterogeneous group of autoimmune diseases characterized by chronic muscle inflammation often accompanied by other extra-muscular inflammation or involvement. Muscle weakness, myalgia, and impaired endurance are common findings among patients. Extra-muscular tissues and organs impacted by IIMs can include, but are not limited to, the lungs, skin, joints, and the heart.^1^ There are several subtypes of IIM, including immune-mediated necrotizing myopathy (IMNM), dermatomyositis (DM), polymyositis (PM), and antisynthetase syndrome (ASyS). Treatment for IIMs typically involves medications to suppress the immune system and reduce inflammation. Although some patients may have only mild symptoms that can be managed with medications, a significant portion of the IIM patient population has disease that requires more aggressive treatment or that remains refractory to existing medications.^2^ Moreover, there are no FDA-approved therapies for IMNM.

Although the pathogenesis of IIM is not well understood, there is evidence that B cells play a pathogenic role in promoting autoantibody formation. Muscle biopsies taken from IIM patients exhibit active inflammatory processes, with membrane attack complex deposition and leukocytic infiltration, antibody secreting cells, transitional B cells with type 1 interferon signatures, and germinal center-like structures.^3^^,^^4^^,^^5^ A total of 70%–80% of patients with IIM have myositis-specific antibodies (MSAs) or myositis-associated antibodies (MAAs), including the three subtypes IMNM, DM, and ASyS, suggesting an autoreactive B cell element in IIM.^6^

A recent meta-analysis of IIM treatment with B cell depleting antibody, rituximab, reported an overall efficacy rate of 65% for all IIM types in a total of 26 global studies.^7^ However, a randomized controlled trial did not show a benefit of rituximab in the treatment of refractory adult PM, or adult or juvenile DM.^8^ Despite therapeutic impact in some patients, rituximab therapy primarily remains non-curative despite prolonged administration over time, potentially due to limited antibody penetrance into lymphoid compartments and incomplete B cell depletion.^9^^,^^10^ Thus, it is possible that deep depletion of B cells in the body for a sufficient period of time in patients with IIM may lead to longer-term disease remission by removing a central driver of inflammation (autoreactive B cells) and allowing the immune system to return to a tolerant state.

Chimeric antigen receptor (CAR) T cells targeting CD19^+^ B cells have been shown to eliminate B cells both in the peripheral blood and within secondary lymphoid organs.^11^ Recently, CD19-CAR T cell therapy has been used to treat patients diagnosed with various autoimmune diseases, including the ASyS subtype of IIM.^12^ Initial clinical and correlative data suggest that CD19-CAR T cell therapy is well tolerated and highly effective in autoimmune disease with many patients in remission following adoptive T cell transfer with up to 29 months of follow-up.^12^^,^^14^^,^^15^^,^^16^ Here, we provide the first report of emerging clinical and translational data from a phase I/II clinical trial (NCT06154252) using an autologous, fully human 4-1BBz anti-CD19-CAR T cell therapy (CABA-201) in one subject with the IMNM subtype of IIM.

## Results

**Figure 1:**
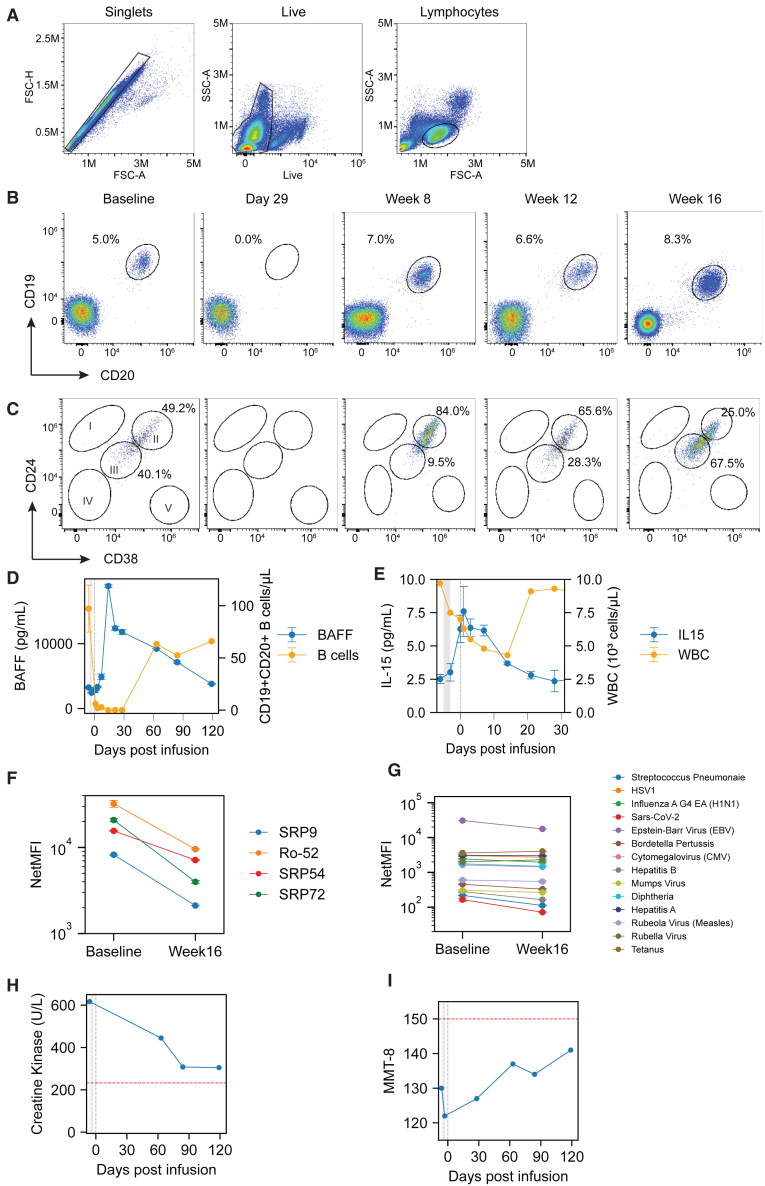
Systemic cellular, humoral, and clinical responses following CABA-201 cell therapy in an INMN subject (A) Gating schema used for B cell phenotype analyses in B, C, and CABA-201 cell phenotype analyses in Figure 2A. (B) B cells identified by flow cytometry at baseline through 16 weeks after CAR T cell infusion. B cells shown in oval gate as the percentage of live, single lymphocytes that are CD19^+^CD20^+^. (C) Maturation status of B cells as determined by flow cytometry at baseline through 16 weeks after CAR T cell infusion as determined by CD24 and CD38 expression. Subpopulation as follows: (I) memory B cells, (II) transitional naive (T1 and T2) B cells, (III) activated naive B cells, (IV) activated naive or activated memory B cells, (V) Plasmablasts or pre-plasmablasts. (D) B cell counts and serum BAFF levels from baseline through 16 weeks post-infusion overlaid as line plots. Left y axis represents serum BAFF levels in pg/mL (blue) and right y axis represents B cells counts as number of CD19^+^CD20^+^ cells/μL blood (orange). The x axis represents time in days from baseline through week 16 with the vertical gray shaded area indicating lymphodepletion in both (D, E, H, and I). (E) Leukocyte counts and serum IL-15 levels overlaid as line plots over time. Left y axis represents serum IL-15 levels in pg/mL (blue) and right y axis represents leukocyte counts as thousand cells/μL blood (orange). Serum levels of myositis-associated autoantibodies (F) and vaccine- or pathogen-associated antibodies (G) at baseline and 16 weeks after CAR T cell infusion. Antibodies measured by Luminex and represented as net median fluorescence intensity. (H) Serum levels of creatine kinase, a marker of skeletal muscle destruction, at baseline through 16 weeks post-infusion. (I) Disease activity assessed by manual muscle testing score 8 (MMT8) at baseline through 16 weeks post-infusion. For (D)–(G) data shown as mean ± SD of duplicate technical replicates.

A 33-year-old male presented with a 23-month history of IMNM, diagnosed based on proximal muscle weakness, elevated creatine kinase (CK) enzymes up to 5,900 U/L, and a positive anti-SRP antibody. The subject was initially treated with corticosteroids along with monthly intravenous immunoglobin and weekly methotrexate. Due to continuing symptoms, the subject was started on rituximab with the last dose approximately 8 months before CABA-201 infusion. Due to continuing symptoms, the patient considered an investigational dose of CABA-201. The last dose of methotrexate 25 mg was 5 days before infusion. At the time of infusion, the subject was taking prednisone 5 mg/day, which was discontinued 3 days after treatment. Immediately prior to infusion, CK still remained elevated at approximately 600 U/L (Figure 1H). Following standard preconditioning regimen with fludarabine and cyclophosphamide, the patient was infused with 1 × 10^6^ CABA-201 CAR T cells per kilogram of weight (a total dose of 8.34 × 10^7^ cells). The infusion was well tolerated. The patient was discharged after 4 days and no cytokine release syndrome (CRS), immune effector cell-associated neurotoxicity syndrome (ICANS), or serious adverse events were reported over the first 4 months following infusion. Over the course of 16 weeks following infusion, a decrease in CK was observed (Figure 1H). Furthermore, a clinical improvement in muscle strength as assessed by the manual muscle test score 8 (MMT8) was also observed (Figure 1I).

### Impact of CABA-201 on the B cell compartment and associated serologic changes

Following CABA-201 infusion, circulating B cells decreased rapidly and were undetectable by day 15 after infusion and remained undetectable through day 29 (Figures 1B and 1D). B cell reduction was accompanied by an increase in serum B cell-activating factor (BAFF) (Figure 1D). B cells returned to the peripheral circulation at approximately 8 weeks following infusion (Figures 1B–1D). Notably, returning B cells were almost entirely transitional naive B cells (Figure 1C). At 12 and 16 weeks post-infusion, circulating B cells were a mix of naive and transitional naive (Figure 1C). B cell recrudescence was associated with a decrease in circulating BAFF (Figure 1D). A transient reduction in peripheral leukocytes resulting from preconditioning was also observed (Figure 1C). Leukocytes returned to pre-infusion levels by day 22 post-infusion. Concurrent with a decrease in peripheral leukocytes, serum IL-15 increased, with a peak observed at day 15, before returning to pre-infusion levels by day 22. At 16 weeks post-infusion, decreases in the serum MSA SRP-9, SRP-54, and SRP-72 and the MAA Ro-52 were observed relative to baseline (Figure 1F). The level of reduction in serum autoantibodies was approximately 74%, 54%, 81%, and 70%, respectively. Of the 14 infectious disease or vaccine-associated antibodies evaluated before and after infusion, no significant changes were observed following infusion, which suggests that pre-existing humoral immunity is not impaired (Figure 1G).

### CABA-201 pharmacokinetics and pharmacodynamics

**Figure 2:**
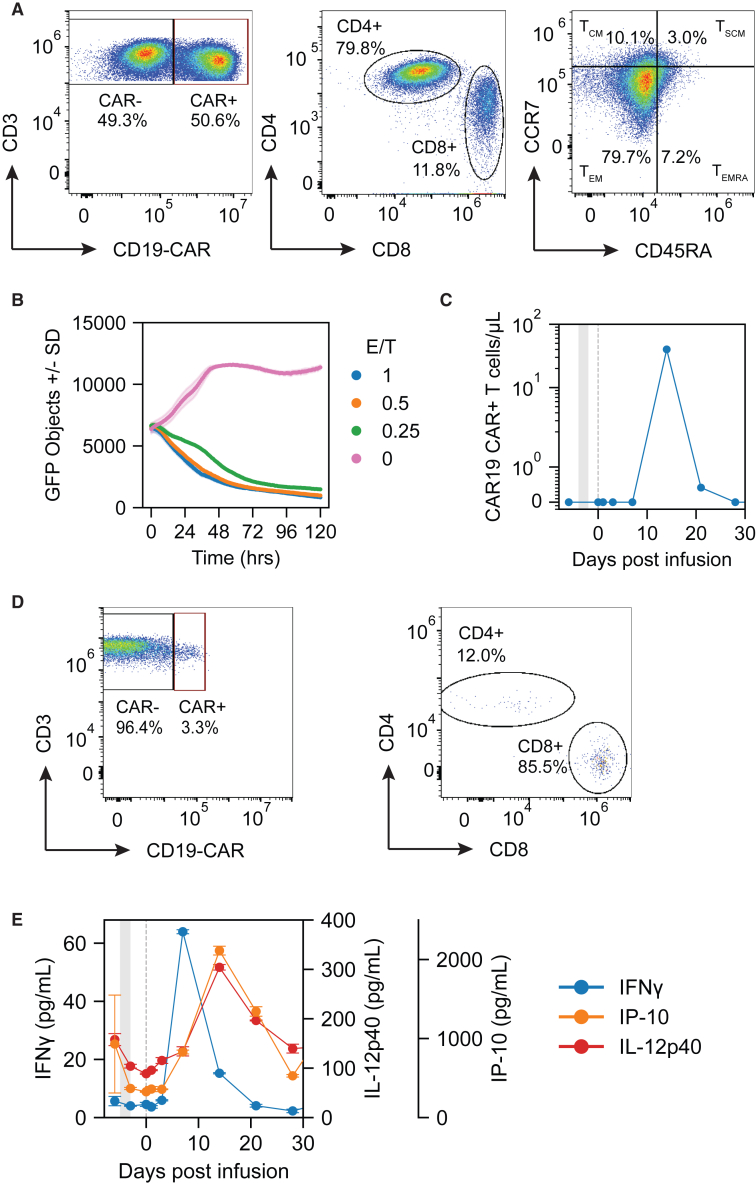
CABA-201 drug product characterization and post-infusion pharmacokinetics and pharmacodynamics (A) Flow cytometric characterization of drug product. Top left: CAR^+^ (right gate) and CAR^−^ (left gate) T cells as a percentage of total CD3^+^ T cells. Top middle: CD4^+^ (y axis) versus CD8^+^ (x axis) CAR^+^ T cells. Top right: memory subsets of CAR T cells defined by CCR7 (y axis) and CD45RA (x axis). Populations represented as the percentage of CAR^+^ T cells that are T_EM_ (CD45RA^–^CCR7^–^), T_EMRA_ (CD45RA^+^CCR7^–^), T_CM_ (CD45RA^–^CCR7^+^), and T_SCM_ (CD45RA^+^CCR7^+^). (B) CABA-201 infusion product lysis of CD19^+^GFP^+^ target NALM6 cells. Cell lysis curves represented by number of GFP^+^ target cells over 120 h at effector to target ratios ranging from 0:1 to 1:1. (C) Pharmacokinetic response of CABA-201 in IMNM subject represented as number of CAR T cells/μL blood from baseline through 29 days post-infusion. (D) Flow cytometric characterization of CABA-201 at day 15. (E) Concentrations of IFN-γ, IP-10 (CXCL10), and IL-12p40 before and after CABA-201 infusion.

The CABA-201 infusion product consisted of 50.6% CAR-positive T cells (Figure 2A) that were 79.3% CD4^+^ and 11.4% CD8^+^; 79.7% of the cells were effector memory (CD45RA^−^CCR7^−^) phenotype (Figure 2A). CABA-201 exhibited cytolytic activity against CD19^+^ NALM6 target cells *in vitro* (Figure 2B). Post-infusion, CAR T cells were detected by digital polymerase chain reaction (dPCR) in the peripheral blood at days 15 and 22 (Figure 2C). Peak expansion of the CAR T cells was calculated to be 40 cells/μL of blood. Furthermore, at peak expansion, approximately 3.3% of all circulating T cells were CAR^+^ (Figure 2D). In contrast to the CABA-201 infusion product, the circulating CAR T cells at day 15 were primarily CD8^+^ (Figure 2D). Serum cytokine profiling revealed elevations in IFN-γ preceding CAR T cell expansion in the peripheral blood (Figure 2E). Serum levels of IP-10 (CXCL10) and IL-12p40 peaked on day 15, concurrent with peak CAR T persistence. Notably, there were no substantial changes in IL-6, IL-8, and GM-CSF post-infusion (data not shown).

## Discussion

This first known report of CD19-CAR T treatment in an IMNM subject adds to the growing body of literature demonstrating the tolerability of CAR T cells for treating autoimmune disease.^13^^,^^16^^,^^17^ The data presented herein provides greater insight into the mechanism of action of 4-1BBζ CD19-CAR T cells in autoimmune disease. To our knowledge, this is the first published report in an IMNM patient, and also the first data reported using CD19-CAR T cells in a phase I/II myositis clinical trial (NCT06154252). A previous report described the use of BCMA directed CAR T cells in IMNM.^18^ Patients with SRP autoantibody IMNM typically have significant muscle weakness often accompanied by myalgia, dyspnea, dysphagia, and muscle atrophy. Standard therapies include glucocorticoids, intravenous immunoglobulin, and B cell-depleting antibody-based therapies.^19^ Although these therapies can benefit patients with IMNM, not all patients respond.^20^ Only half of all SRP autoantibody IMNM patients have satisfactory outcomes with immunotherapy after 4 years.^19^ The subject described in this report had IMNM refractory to several lines of immunosuppressive therapy and was then infused with CABA-201.

We observed a rapid and deep CD19^+^ B cell depletion following CABA-201 administration that occurred simultaneously with CABA-201 expansion (Figures 1B, 1D, and 2C). Furthermore, the B cell depletion was accompanied by an increase in serum BAFF levels (Figure 1D), which has been observed as a homeostatic response to B cell depletion.^21^ BAFF is produced by a wide variety of different cell types including monocytes, macrophages, dendritic cells, epithelial cells, and neutrophils.^22^ As expected, the number of peripheral leukocytes reached its nadir at day 15 and recovered by day 22 after CABA-201 infusion. Given that B cells remained undetectable from day 15 through day 29, these data further support the selective elimination of B cells by CABA-201. B cell repopulation in the peripheral circulation was first observed at 8 weeks post-infusion (Figures 1B–1D). Interestingly, the re-emerging population was almost entirely early, naive transitional CD24^++^ CD38^++^ B cells (Figure 1C). These transitional B cells can be considered recent emigrants out of the bone marrow requiring maturation in the periphery. Furthermore, these cells are unlikely to be autoreactive as they have yet to encounter antigen.^23^

At 4 months following infusion, we observed a decrease in autoantibodies without a decrease in vaccine or infectious disease-associated antibodies in agreement with other reports of autoimmune disease patients treated with CD19-CAR T cells (Figures 1F and 1G).^13^^,^^21^ The reduction of autoantibody levels suggest that autoantibody-producing cells are primarily CD19^+^ or short-lived CD19^–^ plasma cells, while the majority of infectious disease- and vaccine-associated antibodies are likely secreted by CD19^–^ long-lived plasma cells in the bone marrow or other secondary lymphoid tissue.^17^ Consistent with a drop in autoantibodies, a decrease in CK and clinical improvement in muscle strength was achieved following infusion (Figures 1H and 1I). Furthermore, the subject is off all other therapies as of this report. These efficacy data are consistent with previous reports of CD19 CAR T cells in the anti-synthetase subtype of myositis and of BCMA CAR T cells in IMNM.^17^^,^^18^^,^^24^ As both BCMA- and CD19-targeting CAR T cell therapies become more widely utilized across autoimmune diseases, one should consider the role of long-lived humoral immunity. In hematologic malignancies, BCMA CAR T cell therapies also target long-lived plasma cells that underlie humoral immunity to vaccine-preventable infections.^25^ The risk of infection from vaccine-preventable infections should be considered against the potential benefit of either a CD19- or BCMA-directed approach.

Following CABA-201 infusion, serum IFN-γ levels peaked prior to detection of CABA-201 in the peripheral blood (Figures 2C and 2E). The likely hypothesis is that CABA-201 rapidly traffics out of the peripheral blood and becomes activated following contact with CD19^+^ B cells residing in secondary lymphoid tissues. Given the kinetics, the elevations in IP-10 (CXCL10) and IL-12p40 are likely produced in response to IFN-γ secreted by CABA-201. Both cytokines can be secreted by myeloid and dendritic cells found in secondary lymphoid tissues and are elevated post-CAR T cell infusion in hematologic malignancies.^26^^,^^27^ The elevations in IFN-γ, IL-10, and IL-12p40 post-CAR T cell infusion in this myositis subject are relatively modest as compared with what has been reported in hematologic malignancies.^28^^,^^29^ This likely reflects a function of the overall lower target CD19^+^ cell number in autoimmune disease as compared with B cell malignancies and the incorporation of 4-1BB co-stimulatory domain, as opposed to a CD28 co-stimulatory domain, in CABA-201,^30^ factors that also potentially confer a safer profile for the product. This hypothesis is supported by the absence of CRS and ICANS in this subject is consistent with the prior reports of CD19-directed 4-1BBζ CAR T cells in patients with autoimmune diseases.^13^

The pharmacokinetics of CABA-201 differs slightly from other reports of CD19 directed 4-1BBζ CAR T cells. Although the absolute level of peak expansion of 40 cells/μL is within the range of what has been reported across autoimmune diseases, the timing of the peak is approximately 7 days later (Figure 2C).^12^^,^^24^^,^^31^ A plausible explanation for the difference in kinetics is that the CABA-201 infusion product is frozen post-manufacture and is thawed immediately before infusion, whereas published literature with other CD19 4-1BBζ CAR T infusion products given to autoimmune disease patients have been infused fresh without a freeze/thaw step post-manufacture.^12^^,^^24^^,^^31^ Interestingly, at peak expansion, most of the CAR T cells in the peripheral blood were CD8^+^ in contrast to the CD4^+^ dominant infusion product (Figures 2A and 2D). This is consistent with previous reports showing CD8^+^ CAR T cells outcompeting CD4^+^ CAR T cells.^32^^,^^33^ An alternative hypothesis is that CD4^+^ and CD8^+^ CAR T cells have differing trafficking patterns. Without performing lymph node biopsies, it is impossible to determine if altered trafficking exists.

In summary, our data provide mechanistic insights into the activity of CABA-201, a fully human CD19 4-1BBζ CAR T, in IMNM. We show that CABA-201 was well tolerated, can rapidly eliminate target CD19^+^ B cells post-infusion, and reduce disease-associated autoantibodies without impacting pre-existing humoral immunity 4 months after infusion. Furthermore, we can better define the pharmacodynamics of CABA-201 infusion, namely that CD19 4-1BBζ CAR T activity can be assessed both by depletion of peripheral B cells and in elevations in serum IFN-γ prior to expansion of T cells in the periphery. These findings demonstrate the potential of CABA-201 for IMNM that is refractory to several therapies including targeted CD20 B cell depletion. Based on these encouraging data in the context of a phase I/II trial, treating additional IIM patients, including IMNM patients, is warranted.

## Materials and methods

### Study design and participants

Eligible subjects had a clinical diagnosis of IIM, based on the 2017 The European League Against Rheumatism/American College of Rheumatology classification criteria. Diagnosis of DM, ASyS, or IMNM was based on the presence of serum MSAs. Furthermore, subjects had to have evidence of active disease, despite prior or current treatment with standard of care treatments, as defined by the presence of elevated CK, DM rash, or active disease on muscle biopsy, MRI, or electromyography and the presence of muscle weakness.

The clinical trial (NCT06154252) is a phase I/II, multi-center, open-label study. The primary objective is to evaluate the safety and tolerability of CABA-201 in subjects with active IIM over 28 days post-infusion. Selected secondary objectives include, but are not limited to, evaluating the effects of CABA-201 on peripheral B cell counts, peripheral leukocyte counts, IIM serology, and IIM disease activity. MMT8, a clinical measure of muscle strength, was determined as described previously.^34^ Relevant secondary and exploratory objectives include evaluating the presence and phenotype of CABA-201 cells post-infusion and the activity of CABA-201 cells post-infusion.

In brief, following the identification and consent of eligible subjects in accordance with the protocols approved by the institutional review board at the University of California Irvine School of Medicine, subjects undergo leukapheresis 4–12 weeks before infusion for collection of peripheral blood mononuclear cells (PBMCs). Following CABA-201 manufacture and infusion, subjects were followed for 29 days post-CABA-201 infusion for safety and scheduled research assessments. Serum and PBMCs were collected at baseline (prior to lymphodepletion, 4 ± 2 days before infusion), pre-infusion (after lymphodepletion, 2 ± 2 days before infusion) and at 1 ± 2 days, 4 ± 2 days, 7 ± 2 days, 14 ± 2 days, 21 ± 2 days, 28 ± 2 days, 8 ± 1 weeks, 12 ± 1 weeks, and 16 ± 1 weeks after CABA-201 infusion.

### CABA-201 manufacturing and dosing

CABA-201 cells were manufactured using a previously reported protocol.^26^ In brief, T cells were selected/activated using anti-CD3/CD28 microbeads (Gibco) at a bead/cell ratio of 3:1. Lentiviral vector that carries target CAR was added within 24 h after activation and T cells were expanded in the Xuri bioreactor (Cytiva). The harvested cells were resuspended in the final formulation reagent CryoStor B/CryoStor 10 (BioLife Solutions), and then formulated into cryobags as drug product as well as into vials for correlative tests. The drug product was then frozen to −90°C using a controlled rated freezer and stored at ≤ −130°C. Patients received a preconditioning regimen consisting of fludarabine (25 mg/m^2^ on days −5, −4, and −3) and cyclophosphamide (1,000 mg/m^2^ on day −3) before a single infusion of CABA-201 at 1 × 10^6^ cells/kg.

### Flow cytometry

PBMCs were labeled with anti-human antibodies and reagents to measure B cells and CAR T cell phenotype. B cell detection was performed using anti-CD19 (PE-Fire 700, BioLegend), anti-CD20 (Brilliant Violet 570, BioLegend), anti-CD24 (APC-Cy7, BD Biosciences), anti-CD38 (BV711, BioLegend), and Fixable Viability Dye eFluor 506 (Thermo Fisher Scientific). CAR T cell detection was performed with anti-CD3 (APC-Vio770, Miltenyi), anti-CD4 (PerCP-Vio700, Miltenyi), anti-CD8 (APC, Miltenyi), anti-CCR7 (PE-Vio770, Miltenyi), anti-CD45RA (VioBlue, Miltenyi), Fixable Viability Dye eFluor 506 (Thermo Fisher Scientific), and CD19-CAR detection reagent (Miltenyi Biotech). PBMCs were incubated with fluorescently labeled antibodies for 30 min at 4°C in the dark. For tests including CAR T cell detection reagent, samples were washed and incubated with secondary antibody for 30 min. Labeled samples were analyzed on the Novocyte Quanteon flow cytometer (Agilent) and gated using FlowJo software (FlowJo).

### Serum cytokine assessment

Serum cytokine levels were quantified using Meso Scale Discovery electrochemiluminescence multiplex platform. Serum samples were tested in duplicate using the VPLEX pro-inflammatory panel, cytokine panel 1, chemokine panel 1, and a custom UPLEX measuring BAFF. Serum samples were tested following the manufacturer’s recommended protocol at a 1:4 and 1:20 dilutions for the VPLEX and UPLEX assays, respectively. Serum cytokine concentrations are reported in pg/mL.

### Leukocyte counts and CK levels

Leukocyte counts were measured by clinical complete blood count test run locally at the clinical site. CK levels were measured by immune assay run locally at the clinical site.

### Serum antibody assessment

Serum antibody titers were quantified using Luminex FlexMap technology (Luminex). Custom Luminex assays were used to measure 24 myositis-associated antibodies and 14 antibodies to infectious diseases, pathogens, and vaccines.^21^ Serum samples were incubated in duplicate with antigen-bound beads and analyzed using a Luminex FlexMap instrument. Antibody reactivity values are represented as net median fluorescence intensity.

### Cytolytic analysis

Cryopreserved drug product was thawed, washed, and rested at 37°C overnight. CD19-CAR T cells were mixed with clonal Nalm6 cells expressing CD19 and green fluorescent protein (GFP) at various effector to target cell ratios in triplicate (1:1, 0.5:1, 0.25:1, and 0:1 or target only). Mixed cell populations were incubated for 5 days at 37°C in the Incucyte live cell imaging platform (Sartorius) with five images per well taken every hour. The number of GFP^+^ target cells were enumerated from images and reported as averages over the 120-h incubation.

### dPCR

Peripheral CAR T cells were quantified using dPCR. DNA was extracted from PBMCs using the QIAamp DNA Blood Mini Kit (QIAGEN) following the manufacturer’s protocol. Following concentration measurement on the Nanodrop One (Thermo Fisher Scientific) samples were diluted to 30 ng/μL in TE buffer. Nanoplate-based dPCR was performed on a QIAcuity Four instrument (QIAGEN ) in a duplex assay measuring IC78 (CAR sequence; F: ACCAAGGTCACCGTCCTA; R: GCTGTATCCAGAACCCTTACAG; P: TTTCACCTCTGCTCCAGACTGCAC) and RPP30 (endogenous control; F: CCAAGAAAGCCAAGTGTGAG; R: TTTGTTGTGGCTGATGAACTAT; P: TGTCAGCACCCTTCTTCCCTTT). DNA (150 ng) was analyzed per reaction; samples were analyzed in triplicate. dPCR thermal cycling parameters were 2 min at 95°C, 40 cycles of 95°C for 15 s, and 62°C for 30 s. Results less than 1 copy/μL were reported if found to be statistically different from the background signal of the assay.^35^ Copy number of IC78 per μg of DNA was calculated for each sample, normalized to RPP30.^36^

## Data and code availability

Data can be made available upon request.

## Acknowledgments

The authors gratefully thank Gwendolyn Binder and Tania Gonzalez Rivera for helpful discussions and proofreading the manuscript. They also gratefully acknowledge Darshil Patel and Chien-Chung Chen for contributing to assay development.

## Author contributions

J.V., D.N., and S.B. designed the research. J.S., M.W., Z.V., A.E., J.W., and J.C. developed assays and performed the experiments. Q.L., T.F., and C.S. oversaw logistics and project management. T.M., C.M., C.L., and D.C. designed the clinical protocol and managed patients. J.S., M.W., Z.V., A.E., J.W., J.C., F.H.-N., and D.T. analyzed the data. D.N., J.V., and S.B. drafted the manuscript. All authors reviewed and interpreted the data and revised the manuscript.

## Declaration of interests

J.V., D.N., J.S., M.W., Z.V., A.E., J.W., J.C., Q.L., T.F., C.S., F.H.-N., D.T., C.M., C.L., D.C., and S.B. are employees of Cabaletta Bio.
